# 5-(4-Hexyl-1*H*-1,2,3-triazol-1-yl)-2,1,3-benzoxadiazole

**DOI:** 10.1107/S1600536812041815

**Published:** 2012-10-13

**Authors:** Jessie A. Key, Christopher W. Cairo, Michael J. Ferguson

**Affiliations:** a Alberta Glycomics Centre, Department of Chemistry, University of Alberta, Edmonton, AB T6G 2G2, Canada; b X-ray Crystallography Laboratory, Department of Chemistry, University of Alberta, Edmonton, AB T6G 2G2, Canada

## Abstract

The title compound, C_14_H_17_N_5_O, a 1,2,3-triazole derivative of benzoxadiazole (C_14_H_17_N_5_O), was synthesized *via* Cu-catal­ysed azide–alkyne cyclo­addition (CuAAC) from the corres­ponding *n*-octyne and 4-azido­benzoxadiazole. The benz­oxa­diazole and triazole rings show a roughly planar orientation [dihedral angle between the ring planes = 12.18 (5)°]. The alkane chain adopts a zigzag conformation, which deviates from the central triazole ring by 20.89 (6)°. These two torsion angles result in an overall twist to the structure, with a dihedral angle of 32.86 (7)° between the benzoxadiazole group and the hexyl chain. The crystal structure features C—H⋯N hydrogen bonds leading to chains propagating along [2-10] and offset parallel stacking inter­actions of the triazole and benzoxadiazole rings. The centroid of the extended π-system formed by the benzoxadiazole and triazole rings (14 atoms total) was calculated; the centroid–centroid distance was 4.179 Å, interplanar separation was 3.243 Å, and the resulting offset was 2.636 Å.

## Related literature

For the synthesis of the title compound and related benz­oxa­diazole analogs, see: Key & Cairo (2011 ▶). For computational studies of the absorption and fluorescence properties of this series of compounds, see: Brown *et al.* (2012 ▶). For structures with 1-aryl-substituted 1,2,3-triazole rings, see: Costa *et al.* (2006 ▶). For the use of fluoro­phores as chemical or biological probes, see: Cairo *et al.* (2010 ▶); Lavis & Raines (2008 ▶). For related benzoxadiazole structures, see: Key *et al.* (2012*a* ▶,*b* ▶). For triazole-substituted coumarin derivatives, see: Key *et al.* (2009 ▶).
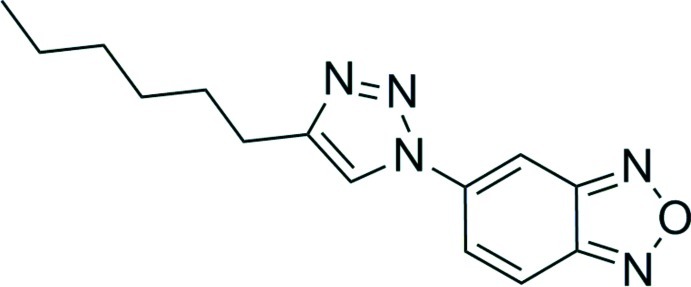


## Experimental

### 

#### Crystal data

C_14_H_17_N_5_O
*M* *_r_* = 271.33
Triclinic, 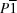

*a* = 5.3604 (8) Å
*b* = 7.8585 (11) Å
*c* = 16.357 (2) Å
α = 87.4656 (17)°
β = 86.2519 (16)°
γ = 85.6240 (17)°
*V* = 685.04 (17) Å^3^
*Z* = 2
Mo *K*α radiation
μ = 0.09 mm^−1^
*T* = 173 K
1.02 × 0.35 × 0.03 mm

#### Data collection

Bruker APEXII CCD diffractometer
Absorption correction: multi-scan (*SADABS*; Bruker, 2008 ▶) *T*_min_ = 0.915, *T*_max_ = 0.997
6114 measured reflections
3120 independent reflections
2568 reflections with *I* > 2σ(*I*)
*R*_int_ = 0.013

#### Refinement

*R*[*F* ^2^ > 2σ(*F* ^2^)] = 0.036
*wR*(*F* ^2^) = 0.099
*S* = 1.04
3120 reflections
181 parameters
H-atom parameters constrained
Δρ_max_ = 0.20 e Å^−3^
Δρ_min_ = −0.22 e Å^−3^

### 

Data collection: *APEX2* (Bruker, 2008 ▶); cell refinement: *SAINT* (Bruker, 2008 ▶); data reduction: *SAINT*; program(s) used to solve structure: *SHELXD* (Sheldrick, 2008 ▶); program(s) used to refine structure: *SHELXL97* (Sheldrick, 2008 ▶); molecular graphics: *SHELXTL* (Sheldrick, 2008 ▶); software used to prepare material for publication: *SHELXTL*.

## Supplementary Material

Crystal structure: contains datablock(s) I, global. DOI: 10.1107/S1600536812041815/mw2081sup1.cif

Supplementary material file. DOI: 10.1107/S1600536812041815/mw2081Isup2.cdx

Structure factors: contains datablock(s) I. DOI: 10.1107/S1600536812041815/mw2081Isup3.hkl

Supplementary material file. DOI: 10.1107/S1600536812041815/mw2081Isup4.cml

Additional supplementary materials:  crystallographic information; 3D view; checkCIF report

## Figures and Tables

**Table 1:** Hydrogen-bond geometry (Å, °)

*D*—H⋯*A*	*D*—H	H⋯*A*	*D*⋯*A*	*D*—H⋯*A*
C3—H3⋯N2^i^	0.95	2.52	3.4674 (15)	177
C5—H5⋯N4^ii^	0.95	2.46	3.3445 (15)	154

Symmetry codes: (i) 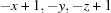; (ii) 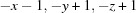.

**Fig. 1.**
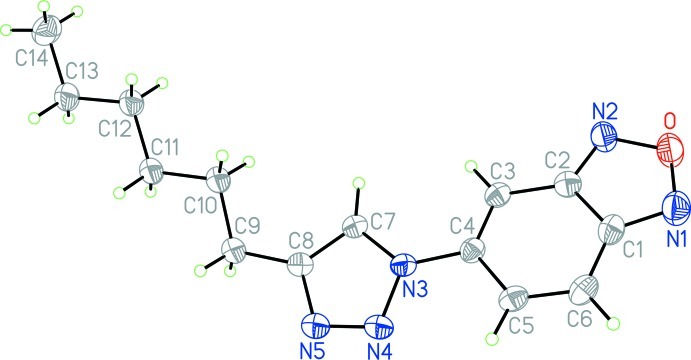
: View of I. Non-hydrogen atoms are represented by Gaussian ellipsoids at the 50% probability level.

**Fig. 2.**
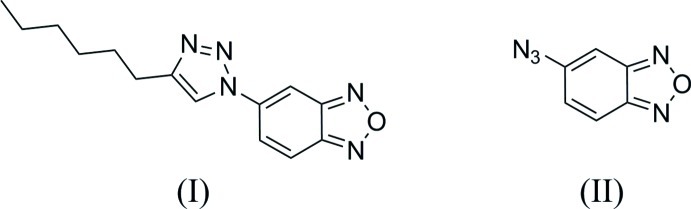
: Compounds used in this study.

**Fig. 3.**
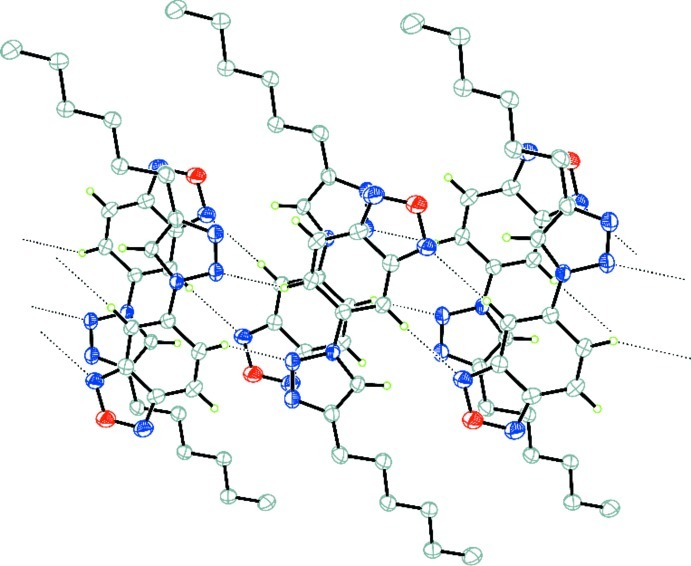
: Packing of I, showing the offset stacking of the triazole and benzoxadiazole rings. C–H···N hydrogen bonds are indicated by dotted lines.

**Fig. 4.**
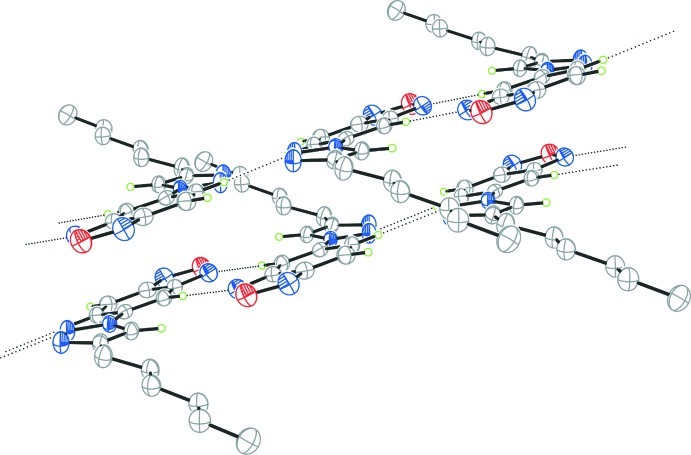
: Alternate view of the packing of I.

## supplementary crystallographic information

### Comment

Fluorophores with properties responsive to their chemical environment, or which are reactive to the presence of specific functional groups, can be useful probes in chemistry and biology (Cairo *et al.*, 2010; Lavis & Raines, 2008). We explored a series of analogs suitable for the Cu-catalyzed azide-alkyne cycloaddition (CuAAC) to identify substrates which were fluorogenic. Compounds with increased fluorescence upon conversion from either an azide or alkyne precursor to a triazole product would be of interest for detecting the presence of these functional groups in complex mixtures. The title compound, I, was found to be only weakly fluorescent, which is in contrast to the properties of the 4-azido-benzoxadiazole precursor, II. Thus, we designated compound I as a quenched fluorophore (Key & Cairo, 2011).

The benzoxadiazole and triazole rings of I are nearly eclipsed, as noted from the dihedral angle of 12.18 (5)° that was obtained from least-squares planes calculations. The hexyl side-chain adopted an essentially planar zigzag conformation [maximum deviation from the plane 0.0530 (8) Å]; the alkyl chain was twisted 20.89 (6)° with respect to the central triazole. There was an overall twist to the structure, with an angle of 32.86 (7)° between the benzoxadiazole group and the hexyl chain.

The packing of I in the solid state was stabilized by weak intermolecular C–H···N hydrogen bonds (H···N distances: 2.46-2.52 Å) and offset parallel stacking interactions (3.331-3.791 Å) of the triazole and benzoxadiazole rings (see Figures 3 and 4).

### Experimental

4-Azidobenzoxadiazole (30 mg, 0.19 mmol, 1 equiv) was dissolved in 1:1 water/methanol (5 mL), followed by addition of *n*-octyne (0.14 mL, 0.93 mmol, 5 equiv). Copper sulfate (6 mg, 0.037 mmol, 0.2 equiv) and ascorbic acid (10 mg, 0.056 mmol, 0.3 equiv) were then added to the mixture. The reaction was allowed to stir at room temperature for 6 h. The reaction was quenched with distilled water, and the crude product was extracted with chloroform, followed by water and brine washes. The organic layer was then dried over MgSO_4_ and concentrated *in vacuo*. Purification was performed by column chromatography (EtOAc/hexanes). The product was obtained as white crystals (25 mg, 50%). m.p. 113.2–115.4 °C; ^1^H NMR (400 MHz, CDCl_3_): δ 8.19 (dd, 1H, ^4^J = 3.8 Hz, ^4^J = 1.84 Hz), 8.06 (m, 2H), 7.91 (s, 1H), 2.85 (t, 2H, ^3^J = 8.0 Hz), 1.77 (m, 2H), 1.31–1.47 (m, 6H),0.91 (m, 3H); ^13^C NMR (100 MHz, CDCl_3_): δ150.5, 149.0, 148.4, 139.1, 126.8, 119.1, 118.9, 104.5, 31.8, 29.4, 29.1, 25.9, 22.8, 14.3; IR (microscope): ν = 3147, 3114, 3095, 3059, 2956, 2929, 2857, 1637, 1540 cm^-1^; ES-HRMS calculated for C_14_H_18_N_5_O [*M*+H]: 272.1506; observed: 272.1513. *R*_f_ = 0.51 (1:3 EtOAc/hexanes).

### Refinement

Although the hydrogen atoms could have been discerned in the difference electron density map, all H atoms were generated in idealized positions and refined using a riding model with fixed C—H distances (C_aryl_ = 0.95 Å, C_methyl_ = 0.98 Å, C_methylene_ = 0.99 Å) and with *U*_iso_(H) = 1.2*U*_eq_(C).

### Crystal data

**Table:**

C_14_H_17_N_5_O	*Z* = 2
*M**_r_* = 271.33	*F*(000) = 288
Triclinic, *P*1	*D*_x_ = 1.315 Mg m^−^^3^
Hall symbol: -P 1	Mo *K*α radiation, λ = 0.71073 Å
*a* = 5.3604 (8) Å	Cell parameters from 5179 reflections
*b* = 7.8585 (11) Å	θ = 2.5–27.5°
*c* = 16.357 (2) Å	µ = 0.09 mm^−^^1^
α = 87.4656 (17)°	*T* = 173 K
β = 86.2519 (16)°	Plate, colourless
γ = 85.6240 (17)°	1.02 × 0.35 × 0.03 mm
*V* = 685.04 (17) Å^3^	

### Data collection

**Table:**

Bruker APEXII CCD diffractometer	3120 independent reflections
Radiation source: fine-focus sealed tube	2568 reflections with *I* > 2σ(*I*)
Graphite monochromator	*R*_int_ = 0.013
ω scans	θ_max_ = 27.6°, θ_min_ = 2.5°
Absorption correction: multi-scan (*SADABS*; Bruker, 2008)	*h* = −6→6
*T*_min_ = 0.915, *T*_max_ = 0.997	*k* = −10→10
6114 measured reflections	*l* = −21→21

### Refinement

**Table:**

Refinement on *F*^2^	Primary atom site location: structure-invariant direct methods
Least-squares matrix: full	Secondary atom site location: difference Fourier map
*R*[*F*^2^ > 2σ(*F*^2^)] = 0.036	Hydrogen site location: inferred from neighbouring sites
*wR*(*F*^2^) = 0.099	H-atom parameters constrained
*S* = 1.04	*w* = 1/[σ^2^(*F*_o_^2^) + (0.0495*P*)^2^ + 0.1216*P*] where *P* = (*F*_o_^2^ + 2*F*_c_^2^)/3
3120 reflections	(Δ/σ)_max_ = 0.002
181 parameters	Δρ_max_ = 0.20 e Å^−^^3^
0 restraints	Δρ_min_ = −0.22 e Å^−^^3^

### Special details

**Table:**

Geometry. All e.s.d.'s (except the e.s.d. in the dihedral angle between two l.s. planes) are estimated using the full covariance matrix. The cell e.s.d.'s are taken into account individually in the estimation of e.s.d.'s in distances, angles and torsion angles; correlations between e.s.d.'s in cell parameters are only used when they are defined by crystal symmetry. An approximate (isotropic) treatment of cell e.s.d.'s is used for estimating e.s.d.'s involving l.s. planes.
Refinement. Refinement of *F*^2^ against ALL reflections. The weighted *R*-factor *wR* and goodness of fit *S* are based on *F*^2^, conventional *R*-factors *R* are based on *F*, with *F* set to zero for negative *F*^2^. The threshold expression of *F*^2^ > σ(*F*^2^) is used only for calculating *R*-factors(gt) *etc*. and is not relevant to the choice of reflections for refinement. *R*-factors based on *F*^2^ are statistically about twice as large as those based on *F*, and *R*- factors based on ALL data will be even larger.

### Fractional atomic coordinates and isotropic or equivalent isotropic displacement parameters (Å^2^)

**Table:**

	*x*	*y*	*z*	*U*_iso_*/*U*_eq_	
O	0.41878 (17)	0.07628 (11)	0.71631 (5)	0.0427 (2)	
N1	0.2106 (2)	0.18420 (14)	0.73699 (6)	0.0420 (3)	
N2	0.44587 (19)	0.04944 (13)	0.63299 (6)	0.0359 (2)	
N3	−0.10674 (16)	0.28627 (11)	0.42563 (5)	0.0269 (2)	
N4	−0.33882 (18)	0.36159 (13)	0.41382 (6)	0.0351 (2)	
N5	−0.36564 (18)	0.36862 (13)	0.33489 (6)	0.0370 (2)	
C1	0.1091 (2)	0.22446 (14)	0.66688 (7)	0.0316 (2)	
C2	0.2550 (2)	0.14071 (13)	0.60238 (7)	0.0287 (2)	
C3	0.1873 (2)	0.15961 (13)	0.51960 (6)	0.0278 (2)	
H3	0.2835	0.1041	0.4763	0.033*	
C4	−0.0229 (2)	0.26178 (13)	0.50634 (6)	0.0259 (2)	
C5	−0.1709 (2)	0.34929 (13)	0.57088 (7)	0.0299 (2)	
H5	−0.3147	0.4207	0.5575	0.036*	
C6	−0.1098 (2)	0.33215 (14)	0.64981 (7)	0.0336 (3)	
H6	−0.2082	0.3892	0.6921	0.040*	
C7	0.0134 (2)	0.24553 (14)	0.35268 (6)	0.0284 (2)	
H7	0.1772	0.1920	0.3437	0.034*	
C8	−0.1520 (2)	0.29817 (14)	0.29511 (7)	0.0306 (2)	
C9	−0.1308 (2)	0.29097 (17)	0.20385 (7)	0.0374 (3)	
H9A	−0.2088	0.3989	0.1804	0.045*	
H9B	−0.2280	0.1970	0.1878	0.045*	
C10	0.1352 (2)	0.26410 (16)	0.16610 (7)	0.0343 (3)	
H10A	0.2127	0.1538	0.1872	0.041*	
H10B	0.2354	0.3561	0.1827	0.041*	
C11	0.1402 (2)	0.26410 (15)	0.07277 (7)	0.0340 (3)	
H11A	0.0493	0.1669	0.0568	0.041*	
H11B	0.0490	0.3704	0.0526	0.041*	
C12	0.4022 (2)	0.25125 (16)	0.03056 (7)	0.0340 (3)	
H12A	0.4936	0.3484	0.0462	0.041*	
H12B	0.4938	0.1447	0.0503	0.041*	
C13	0.4010 (2)	0.25185 (16)	−0.06240 (7)	0.0379 (3)	
H13A	0.3132	0.1529	−0.0781	0.045*	
H13B	0.3055	0.3570	−0.0819	0.045*	
C14	0.6621 (3)	0.24356 (19)	−0.10491 (8)	0.0466 (3)	
H14A	0.6491	0.2458	−0.1644	0.056*	
H14B	0.7560	0.1377	−0.0874	0.056*	
H14C	0.7497	0.3418	−0.0903	0.056*	

### Atomic displacement parameters (Å^2^)

**Table:**

	*U*^11^	*U*^22^	*U*^33^	*U*^12^	*U*^13^	*U*^23^
O	0.0479 (5)	0.0486 (5)	0.0312 (4)	0.0037 (4)	−0.0081 (4)	−0.0020 (4)
N1	0.0476 (6)	0.0462 (6)	0.0318 (5)	0.0020 (5)	−0.0039 (5)	−0.0046 (4)
N2	0.0388 (6)	0.0387 (5)	0.0302 (5)	−0.0004 (4)	−0.0060 (4)	−0.0015 (4)
N3	0.0223 (4)	0.0292 (4)	0.0283 (5)	0.0012 (3)	0.0008 (3)	−0.0011 (3)
N4	0.0250 (5)	0.0441 (6)	0.0348 (5)	0.0057 (4)	−0.0011 (4)	−0.0015 (4)
N5	0.0283 (5)	0.0477 (6)	0.0336 (5)	0.0047 (4)	−0.0009 (4)	−0.0003 (4)
C1	0.0374 (6)	0.0312 (6)	0.0266 (5)	−0.0057 (5)	0.0007 (4)	−0.0031 (4)
C2	0.0288 (6)	0.0261 (5)	0.0314 (5)	−0.0036 (4)	−0.0010 (4)	−0.0007 (4)
C3	0.0275 (5)	0.0285 (5)	0.0272 (5)	−0.0017 (4)	0.0023 (4)	−0.0037 (4)
C4	0.0263 (5)	0.0259 (5)	0.0256 (5)	−0.0053 (4)	0.0007 (4)	−0.0010 (4)
C5	0.0279 (6)	0.0280 (5)	0.0328 (6)	0.0004 (4)	0.0040 (4)	−0.0028 (4)
C6	0.0378 (6)	0.0323 (6)	0.0299 (6)	−0.0016 (5)	0.0063 (5)	−0.0062 (4)
C7	0.0241 (5)	0.0334 (6)	0.0268 (5)	0.0008 (4)	0.0018 (4)	−0.0011 (4)
C8	0.0247 (5)	0.0354 (6)	0.0312 (6)	−0.0013 (4)	−0.0007 (4)	0.0013 (4)
C9	0.0299 (6)	0.0539 (7)	0.0275 (6)	0.0017 (5)	−0.0039 (4)	0.0031 (5)
C10	0.0313 (6)	0.0438 (7)	0.0271 (5)	0.0021 (5)	−0.0035 (4)	0.0002 (5)
C11	0.0325 (6)	0.0416 (6)	0.0272 (5)	0.0018 (5)	−0.0043 (4)	0.0010 (4)
C12	0.0333 (6)	0.0410 (6)	0.0274 (5)	0.0009 (5)	−0.0034 (4)	−0.0017 (4)
C13	0.0390 (7)	0.0468 (7)	0.0275 (6)	−0.0001 (5)	−0.0031 (5)	−0.0009 (5)
C14	0.0459 (8)	0.0612 (9)	0.0326 (6)	−0.0065 (6)	0.0041 (5)	−0.0064 (6)

### Geometric parameters (Å, º)

**Table:**

O—N1	1.3821 (14)	C8—C9	1.4929 (15)
O—N2	1.3849 (12)	C9—C10	1.5195 (16)
N1—C1	1.3170 (15)	C9—H9A	0.9900
N2—C2	1.3173 (14)	C9—H9B	0.9900
N3—C7	1.3576 (13)	C10—C11	1.5251 (15)
N3—N4	1.3581 (13)	C10—H10A	0.9900
N3—C4	1.4227 (13)	C10—H10B	0.9900
N4—N5	1.3067 (13)	C11—C12	1.5223 (16)
N5—C8	1.3709 (14)	C11—H11A	0.9900
C1—C2	1.4257 (15)	C11—H11B	0.9900
C1—C6	1.4288 (16)	C12—C13	1.5207 (15)
C2—C3	1.4231 (15)	C12—H12A	0.9900
C3—C4	1.3563 (15)	C12—H12B	0.9900
C3—H3	0.9500	C13—C14	1.5200 (17)
C4—C5	1.4451 (15)	C13—H13A	0.9900
C5—C6	1.3511 (16)	C13—H13B	0.9900
C5—H5	0.9500	C14—H14A	0.9800
C6—H6	0.9500	C14—H14B	0.9800
C7—C8	1.3648 (15)	C14—H14C	0.9800
C7—H7	0.9500		
			
N1—O—N2	112.30 (8)	C10—C9—H9A	108.5
C1—N1—O	104.60 (9)	C8—C9—H9B	108.5
C2—N2—O	104.44 (9)	C10—C9—H9B	108.5
C7—N3—N4	110.32 (9)	H9A—C9—H9B	107.5
C7—N3—C4	129.66 (9)	C9—C10—C11	111.55 (9)
N4—N3—C4	120.02 (9)	C9—C10—H10A	109.3
N5—N4—N3	107.10 (9)	C11—C10—H10A	109.3
N4—N5—C8	109.41 (9)	C9—C10—H10B	109.3
N1—C1—C2	109.28 (10)	C11—C10—H10B	109.3
N1—C1—C6	130.17 (11)	H10A—C10—H10B	108.0
C2—C1—C6	120.55 (10)	C12—C11—C10	114.34 (9)
N2—C2—C3	129.02 (10)	C12—C11—H11A	108.7
N2—C2—C1	109.39 (10)	C10—C11—H11A	108.7
C3—C2—C1	121.58 (10)	C12—C11—H11B	108.7
C4—C3—C2	115.86 (9)	C10—C11—H11B	108.7
C4—C3—H3	122.1	H11A—C11—H11B	107.6
C2—C3—H3	122.1	C13—C12—C11	113.10 (9)
C3—C4—N3	119.77 (9)	C13—C12—H12A	109.0
C3—C4—C5	123.22 (10)	C11—C12—H12A	109.0
N3—C4—C5	117.01 (9)	C13—C12—H12B	109.0
C6—C5—C4	121.65 (10)	C11—C12—H12B	109.0
C6—C5—H5	119.2	H12A—C12—H12B	107.8
C4—C5—H5	119.2	C14—C13—C12	113.29 (10)
C5—C6—C1	117.13 (10)	C14—C13—H13A	108.9
C5—C6—H6	121.4	C12—C13—H13A	108.9
C1—C6—H6	121.4	C14—C13—H13B	108.9
N3—C7—C8	105.12 (9)	C12—C13—H13B	108.9
N3—C7—H7	127.4	H13A—C13—H13B	107.7
C8—C7—H7	127.4	C13—C14—H14A	109.5
C7—C8—N5	108.05 (10)	C13—C14—H14B	109.5
C7—C8—C9	131.26 (10)	H14A—C14—H14B	109.5
N5—C8—C9	120.69 (10)	C13—C14—H14C	109.5
C8—C9—C10	115.02 (9)	H14A—C14—H14C	109.5
C8—C9—H9A	108.5	H14B—C14—H14C	109.5
			
N2—O—N1—C1	−0.10 (12)	C7—N3—C4—C5	167.55 (10)
N1—O—N2—C2	0.04 (12)	N4—N3—C4—C5	−12.02 (14)
C7—N3—N4—N5	−0.01 (12)	C3—C4—C5—C6	−1.06 (17)
C4—N3—N4—N5	179.64 (9)	N3—C4—C5—C6	178.83 (10)
N3—N4—N5—C8	0.00 (13)	C4—C5—C6—C1	0.44 (16)
O—N1—C1—C2	0.11 (12)	N1—C1—C6—C5	−179.58 (12)
O—N1—C1—C6	−179.94 (11)	C2—C1—C6—C5	0.36 (16)
O—N2—C2—C3	−179.31 (10)	N4—N3—C7—C8	0.01 (12)
O—N2—C2—C1	0.03 (12)	C4—N3—C7—C8	−179.59 (10)
N1—C1—C2—N2	−0.09 (13)	N3—C7—C8—N5	−0.01 (12)
C6—C1—C2—N2	179.96 (10)	N3—C7—C8—C9	179.38 (12)
N1—C1—C2—C3	179.30 (10)	N4—N5—C8—C7	0.01 (13)
C6—C1—C2—C3	−0.65 (16)	N4—N5—C8—C9	−179.46 (10)
N2—C2—C3—C4	179.35 (11)	C7—C8—C9—C10	−18.02 (19)
C1—C2—C3—C4	0.08 (15)	N5—C8—C9—C10	161.30 (11)
C2—C3—C4—N3	−179.14 (9)	C8—C9—C10—C11	−178.16 (10)
C2—C3—C4—C5	0.75 (15)	C9—C10—C11—C12	175.60 (10)
C7—N3—C4—C3	−12.56 (16)	C10—C11—C12—C13	−179.91 (10)
N4—N3—C4—C3	167.88 (9)	C11—C12—C13—C14	178.54 (11)

### Hydrogen-bond geometry (Å, º)

**Table:**

*D*—H···*A*	*D*—H	H···*A*	*D*···*A*	*D*—H···*A*
C3—H3···N2^i^	0.95	2.52	3.4674 (15)	177
C5—H5···N4^ii^	0.95	2.46	3.3445 (15)	154

Symmetry codes: (i) −*x*+1, −*y*, −*z*+1; (ii) −*x*−1, −*y*+1, −*z*+1.
